# Microbial seed treatments: An annotated selection of World Wide Web sites relevant to the topics in *microbial biotechnology*

**DOI:** 10.1111/1751-7915.13521

**Published:** 2019-12-19

**Authors:** Lawrence P. Wackett

**Affiliations:** ^1^ Department of Biochemistry, Molecular Biology & Biophysics BioTechnology Institute University of Minnesota St. Paul MN 55108 USA

## Microbial inoculation of seeds: A review

https://www.ncbi.nlm.nih.gov/pmc/articles/PMC4909795/

This journal review article discusses methods and benefits to microbial seed incoulation for introducing beneficial bacteria during seed germination and subsequent plant development.

## Microbe seed treatment for borage

https://seedworld.com/worlds-first-microbial-seed-treatment-developed-for-borage/

This article focuses on business and application facets of creating a microbial seed treatment process for borage. Borage is an herb crop that is an important source of gamma‐linolenic acid.

## Microbial solutions in agriculture: Novozymes

https://www.novozymes.com/en/advance-your-business/agriculture/crop-production

Novozymes is a global leader in industrial‐scale enzymes. This commercial page describes their interests in microbial processes for promoting plant growth and productivity, including seed treatments.

## Microbial treatment of soybean seeds

http://cardycropsolutions.com/uploads/planting_soybeanbiologicalmicrobialseedtreatments.pdf

This commercial article provides a good overview of the overall process of, and developments in, microbial seed coatings. The experimental part focuses on soybean.

## Treating wheat seeds with neonicotinoid insecticides

https://journals.plos.org/plosone/article?id=10.1371/journal.pone.0205200

Neonicotinoid pesticides are increasingly being used to treat seeds to prevent damage from pests such as aphids. This study examined the effect of neonicotinoinds on microbes.

## Agricultural seed treatments: Corteva

https://www.corteva.us/products-and-solutions/seed-treatments.html

This website describes commercial products. In the process, it provides good insights into the types and manner of microbial seed protection.

## Seed treatment system: BASF

https://agriculture.basf.com/us/en/Crop-Protection/Poncho-VOTiVO-2-0.html

This site highlights an ongoing trend of combining an insecticide and a microbe on the same seed for enhanced survival and performance.

## Microbial populations on seeds

https://www.nature.com/articles/s41598-019-42865-9

Seeds are transmitters of microbes into a soil upon planting. The transmission can be for beneficial or detrimental bacteria. This study looked at pathogen transmittal and how the pathogens might be out‐competed by other microbes.

## Science behind microbial plant products

https://www.agriculture.com/crops/the-science-behind-microbial-and-biological-products

This popular science article gives a good overview of this field of research and highlights some of the researchers working in the field.

## Microbial seed treatments: Acceleron

http://www.acceleronsas.com/Solutions/BioEnhancers/Pages/Acceleron-BioAg.aspx

This is a commercial website, but information on the products highlights important features of microbial seed treatments.

## Seed treatment: Syngenta

http://www.syngenta-us.com/crop-protection/seed-treatment

This website highlights the seed treatment products of the agricultural products company Syngenta.

## Encapsulation for microbial seed treatment: Patent

https://patents.google.com/patent/WO2017087939A1/en

This patent describes methods for encapsulating microorganisms in a polymer gel on seeds and the utility of such processes.

## Endophytic microbial seed treatment

https://patentscope.wipo.int/search/en/detail.jsf?docId=US243315432

This patent focuses on formulations, specifically the most effective microbial combinations for particular seed treatments.

## EPA registers soybean seed treatment

https://headsupst.com/crops/soybean-crops/

This page highlights an approval of microbial seed treatment for pest control by a regulatory body, in this case the U.S. Environmental Protection Agency.

## Microencapsulated *Bacillus*

https://www.ncbi.nlm.nih.gov/pmc/articles/PMC4738727/

This publication describes a specific type of seed treament, a particular *Bacillus* strain and it effects on cotton seedlings.

## Application of microorganisms to seeds

https://link.springer.com/content/pdf/10.1007%2F978-94-011-4926-6_8.pdf

This extensive review article is somewhat dated but it provides a good, comprehensive description of many aspects of coating microbes onto seeds.

## Microbiome engineering by seed coating

https://www.frontiersin.org/articles/10.3389/fmicb.2017.00011/full

This study details an approach of applying a benefical microbe to flowers with the effect of ultimately transmitting the microbe to harvested seeds.

## Rhizobacteria in nanofibers

https://journals.plos.org/plosone/article?id=10.1371/journal.pone.0176930

This paper described a method for using electrospinning to nanoimmobilize microbes for subsequent seed applications.

